# Mutations in transcription factors that confer fluconazole resistance also confer reduced susceptibility to manogepix in *Candida auris* (*Candidozyma auris*), *Candida albicans*, *Candida parapsilosis*, and *Candida glabrata* (*Nakaseomyces glabratus*)

**DOI:** 10.1128/aac.00680-25

**Published:** 2025-07-23

**Authors:** Katherine S. Barker, Hoja P. Patterson, Joachim Morschhäuser, Christina A. Cuomo, Nathan P. Wiederhold, P. David Rogers

**Affiliations:** 1 Department of Pharmacy and Pharmaceutical Sciences, St Jude Children's Research Hospital 5417, Memphis, Tennessee, USA; 2 Department of Pathology and Laboratory Medicine, Fungus Testing Laboratory, The University of Texas Health Science Center at San Antonio 14742, San Antonio, Texas, USA; 3 Institute of Molecular Infection Biology, University of Würzburg, Würzburg, Bavaria, Germany; 4 Department of Molecular Microbiology and Immunology, Brown University 6752, Providence, Rhode Island, USA; 5 Broad Institute 33577, Cambridge, Massachusetts, USA; University of Iowa, Iowa City, Iowa, USA

**Keywords:** *Candida glabrata*, *Candida parapsilosis*, *Candida albicans*, *Candida auris*, manogepix, fluconazole

## Abstract

The fungal pathogen *Candida auris* is of global concern due to high levels of multidrug resistance and its propensity to cause infectious outbreaks. Over 90% of isolates are resistant to fluconazole, the most commonly prescribed antifungal worldwide. Fluconazole resistance is multifactorial with many isolates carrying mutations in the gene encoding the transcriptional regulator Tac1B, leading to increased expression of the gene encoding the ATP-binding cassette (ABC) transporter Cdr1. Recently, a study in *C. auris* examining mechanisms of *in vitro*-evolved reduced susceptibility to manogepix, a promising antifungal agent currently in clinical trials, found a *TAC1B* mutation that confers reduced manogepix and fluconazole susceptibility. We hypothesized that mutations in *C. auris TAC1B* and similar transcription factors in other *Candida* species that confer fluconazole resistance might also confer reduced susceptibility to manogepix. We measured manogepix susceptibilities for selected isolates and strains and found that mutations in *C. auris TAC1B*, *C. albicans* and *C. parapsilosis TAC1*, and *C. glabrata PDR1* confer reduced manogepix susceptibility in a manner dependent on ABC transporters, such as Cdr1. Our findings raise the possibility of fluconazole and manogepix cross-resistance for clinical isolates harboring mutations in these genes.

## INTRODUCTION

Invasive candidiasis is associated with significant morbidity and mortality. It is estimated that over 400,000 people die annually from invasive candidiasis (IC) with an average mortality rate of 35% (1). The majority of infections are caused by five *Candida* species: *Candida albicans*, *Candida glabrata* (*Nakaseomyces glabratus*), *Candida tropicalis*, *Candida parapsilosis*, and *Candida krusei* (*Pichia kudriavzevil*) (2). Moreover, *Candida auris* (*Candidozyma auris*) has emerged as a global health threat owing to its propensity to cause outbreaks in the healthcare environment and high rates of antifungal resistance (3, 4). Three classes of antifungal agents are useful for the treatment of IC: the echinocandins, such as micafungin, the triazoles, such as fluconazole, and the polyene amphotericin B. While the echinocandins have emerged as front-line therapy for the treatment of serious infections due to *Candida* species, fluconazole is still widely used and remains the most commonly prescribed antifungal in the United States (5).

Fosmanogepix is a prodrug of the active moiety manogepix, which acts by inhibiting Gwt1, a key enzyme in the glycosylphosphatidylinositol pathway and biosynthesis of glycosylphosphatidylinositol (GPI) anchors, resulting in compromised cell wall integrity (6). With the exception of the less common species *C. krusei, C. inconspicua,* and *C. kefyr*, manogepix exhibits broad-spectrum fungistatic activity against *Candida* species (7). While formal susceptibility breakpoints have not been established, wild-type upper limit values have been determined for manogepix as 0.03 µg/mL for *C. albicans*, 0.06 µg/mL for *C. parapsilosis*, 0.06–0.125 µg/mL for *C. auris*, and 0.125 µg/mL for *C. glabrata* (7, 8). In that study, a correlation with fluconazole susceptibility was observed. One possible explanation is the contribution of efflux pumps as manogepix susceptibility has been shown to be influenced by *CDR11* and *SNQ2* encoding ATP-binding cassette (ABC) transporters in *C. albicans* and *MDR1* encoding a major facilitator superfamily (MFS) transporter in *C. parapsilosis* (9).

In *C. albicans*, a major driver of fluconazole resistance is overexpression of the ABC transporter gene *CDR1* due to activating mutations in the zinc cluster transcription factor *TAC1* (10). In *C. parapsilosis*, resistance is driven in part by overexpression of *CDR1*, *CDR1B*, and *CDR1C*, due to activating mutations in *TAC1*, whereas in *C. auris*, it is driven in part by overexpression of *CDR1* and to a lesser extent *MDR1* due to mutations in *TAC1B* (11–13). In *C. glabrata*, fluconazole resistance is almost exclusively due to activating mutations in the zinc cluster transcription factor gene *PDR1* that lead to overexpression of the *CDR1*, *PDH1*, and *SNQ2* transporter genes alone or in combination (14–16).

Recently, *in vitro* evolution studies in *C. auris* identified a mutation in *TAC1B* in laboratory strains evolved to have reduced susceptibility to manogepix (17). This D865N amino acid substitution resulted in increased expression of *CDR1* and loss of either *CDR1* or *TAC1B* resulted in increased manogepix susceptibility in these strains. These findings suggest that fluconazole-resistant *Candida* isolates with mutations in similar transcription factor genes may likewise affect manogepix susceptibility.

In order to assess the contribution of these genes to manogepix susceptibility and to determine the potential for cross-resistance between fluconazole and manogepix, we determined the susceptibilities to manogepix in clinical isolates and strains from our collection that have mutations in *TAC1B* (*C. auris*), *TAC1* (*C. albicans* and *C. parapsilosis*), and *PDR1* (*C. glabrata*) that drive overexpression of ABC drug transporters in these species and are known to contribute to fluconazole resistance.

## RESULTS

### Mutations in *TAC1B* that confer increased resistance to fluconazole in *C. auris* confer reduced susceptibility to manogepix

**Fig 1:**
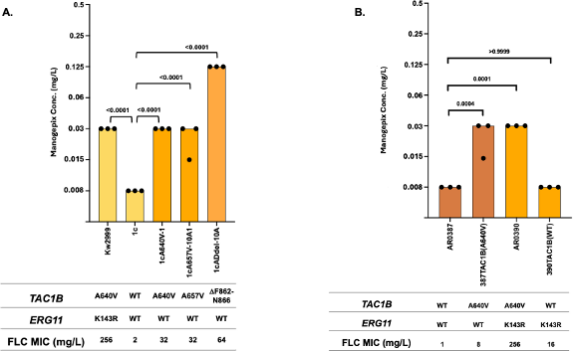
Manogepix MIC for (**A**) *Candida auris* fluconazole-resistant clinical isolate Kw2999 and its derivative strains harboring either *TAC1B*^WT^ (strain 1c) or the most prevalent *TAC1B* mutant alleles, and (**B**) *C. auris* CDC AR Bank isolates AR0387 and AR0390 and their *TAC1B* allele-swapped derivative strains. Each bar in the graph represents the modal MIC value, and each dot represents the measurements from three independent assays.

To determine if mutations in *TAC1B* that confer increased resistance to fluconazole in *C. auris* influence susceptibility to manogepix, we first measured manogepix susceptibilities in the fluconazole-resistant clinical isolate Kw2999, which carries both a mutation leading to the A640V substitution in *TAC1B*, as well as a mutation leading to the K143R substitution in *ERG11*. We also measured susceptibility to manogpix in its fluconazole-susceptible derivative strain 1c where both the *TAC1B* and *ERG11* alleles have been corrected to the wild-type sequences, as well as in derivatives of 1c where mutations in *TAC1B* leading to A640V, A657V, and F862_N866del found in fluconazole-resistant clinical isolates were introduced (Fig. 1A) (13). Isolate Kw2999 exhibited manogepix MICs of 0.03 µg/mL as compared with 0.008 µg/mL for strain 1c, a two-dilution difference in MIC. Introduction of these three mutations into strain 1c resulted in MICs of 0.03, 0.03, and 0.125 µg/mL representing two-, two-, and four-dilution differences in susceptibility.

We then measured manogepix susceptibilities in another fluconazole-susceptible strain AR0387, its *TAC1B*^A640V^ derivative strain, fluconazole-resistant isolate AR0390, which harbors the *TAC1B*^A640V^ allele, and its wild-type *TAC1B* derivative strain (Fig. 1B). Introduction of *TAC1B*^A640V^ into AR0387 reduced manogepix susceptibility by two dilutions from 0.008 to 0.03 µg/mL, whereas its correction to the wild-type sequence in AR0390 reduced manogepix susceptibility by two dilutions from 0.03 to 0.008 µg/mL. These data indicate that activating mutations in *TAC1B* that confer reduced susceptibility to fluconazole in *C. auris* clinical isolates likewise confer reduced susceptibility to manogepix.

### *CDR1* but not *MDR1* drives reduced manogepix susceptibility in fluconazole-resistant isolates of *C. auris* carrying mutations in *TAC1B*

**Fig 2:**
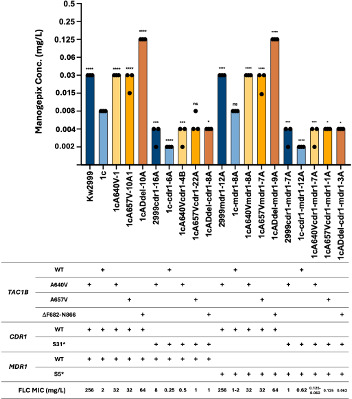
Manogepix MIC for *cdr1*-disrupted, *mdr1*-disrupted, or *cdr1*/*mdr1*-disrupted *TAC1B* mutant *C. auris* strains. Each bar in the graph represents the modal MIC value, and each dot represents the measurements from three independent assays.

In *C. auris*, *TAC1B*-mediated fluconazole resistance is driven by overexpression of both the *CDR1* and *MDR1* transporter genes (13). In isolates that are resistant to fluconazole due to mutations in *TAC1B*, disruption of *CDR1* confers increased fluconazole susceptibility. Disruption of *MDR1* alone has no effect on fluconazole susceptibility, but disruption of both *CDR1* and *MDR1* confers a greater increase in susceptibility than *CDR1* disruption alone. We therefore measured manogepix susceptibilities in a panel of strains carrying mutations in *TAC1B* where *CDR1* and *MDR1* have been disrupted (Fig. 2). Disruption of *CDR1* in strains engineered to express *TAC1B* mutations leading to A640V, A657V, or F862_N866del resulted in a three-, three-, and five-dilution increase in manogepix susceptibility from 0.03, 0.03, and 0.125 µg/mL to 0.004 µg/mL for these strains, respectively. Disruption of *MDR1* in these strains had no effect on manogepix susceptibility, and disruption of both *CDR1* and *MDR1* resulted in no greater increase in susceptibility than *CDR1* disruption alone. These data indicate that *CDR1* is the major driver of reduced manogepix susceptibility due to these *TAC1B* mutations.

### Mutations in *TAC1* that confer increased resistance to fluconazole in *C. albicans* confer reduced susceptibility to manogepix

**Fig 3:**
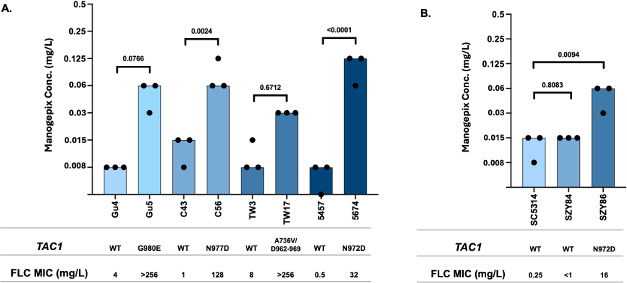
Manogepix MIC for (**A**) *C. albicans* matched set clinical isolates in which the fluconazole-resistant isolate harbors a *TAC1* mutation, and (**B**) *C. albicans* laboratory strain SC5314 and its derivative strains harboring a *TAC1* allele from fluconazole-susceptible isolate 5457 or a *TAC1* allele from fluconazole-resistant isolate 5674. Each bar in the graph represents the modal MIC value, and each dot represents the measurements from three independent assays.

In order to determine if activating mutations in *TAC1* that contribute to fluconazole resistance in *C. albicans* also influence manogepix susceptibility, we determined manogepix susceptibilities in a panel of matched pairs of fluconazole-susceptible and -resistant clinical isolates where the resistant isolates carry activating mutations in *TAC1*. These isolates and strains have been shown previously to overexpress *CDR1* and *CDR2* (18–22). Each resistant isolate was obtained from the same patient as its matched susceptible counterpart over the course of fluconazole treatment. Fluconazole-resistant isolates Gu5, C56, TW17, and 5674 carry mutations leading to the G980E, N977D, A736V/DΔ962-969, and N972D amino acid substitutions, respectively. Their respective manogepix MICs were 0.06, 0.06, 0.03, and 0.125 µg/mL, which is a three-, two-, two-, and four-dilution increase in manogepix MIC relative to their respective matched susceptible isolates (Fig. 3A).

Strains SZY84 and SZY86 are derived from strain SC5314 where either a wild-type TAC1 allele from fluconazole-susceptible clinical isolate 5457 or the *TAC1* allele with a mutation leading to the N972D substitution from resistant isolate 5674 was introduced into a *tac1*Δ/*tac1*Δ strain (22). SZY86 exhibited a two-dilution increase in manogepix MIC to 0.06 µg/mL as compared with 0.015 µg/mL SZY84 (Fig. 3B). These results indicate that activating mutations in *TAC1* that confer fluconazole resistance likewise result in reduced susceptibility to manogepix in *C. albicans*.

### A mutation in *TAC1* that confers increased resistance to fluconazole in *C. parapsilosis* confers reduced susceptibility to manogepix

**Fig 4:**
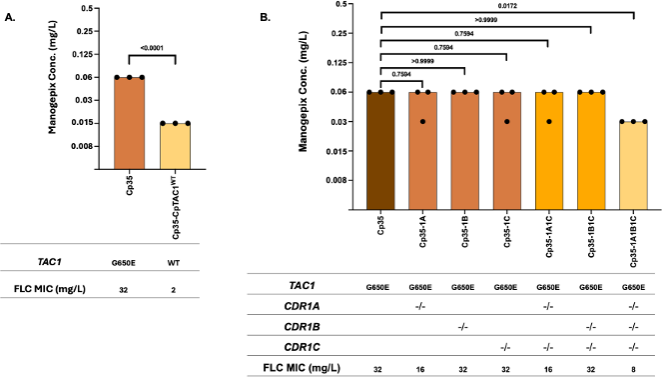
Manogepix MIC for (**A**) *C. parapsilosis* isolate Cp35 and its *TAC1*^WT^ derivative strain, and (**B**) Cp35 and its derivative strains in which *CDR1A*, *CDR1B*, and *CDR1C* have been knocked out individually or in combination. Each bar in the graph represents the modal MIC value, and each dot represents the measurements from three independent assays.

In order to determine if mutations in *TAC1* in *C. parapsilosis* that confer fluconazole resistance influence susceptibility to manogepix, we measured manogepix susceptibilities in clinical isolate Cp35, which harbors a *TAC1*^G650E^ mutation, as well as its derivative where its *TAC1* sequence was corrected to wild-type. Cp35 exhibited a MIC of 0.06 µg/mL, two dilutions higher than its *TAC1*^WT^ derivative (Fig. 4A). This observation indicates that an activating mutation in *TAC1* that confers increased resistance to fluconazole in *C. parapsilosis* likewise confers reduced susceptibility to manogepix.

### Reduced susceptibility to manogepix in *C. parapsilosis* due to mutations in *TAC1* is driven in part by *CDR1A* and *CDR1C*

Fluconazole resistance in *C. parapsilosis* can be due in part to mutations in *TAC1*, which drive the overexpression of *CDR1A*, *CDR1B*, and *CDR1C*. We measured manogepix MICs in the fluconazole-resistant isolate Cp35, which carries a *TAC1*^G650E^ mutation, and its derivatives disrupted for *CDR1A*, *CDR1B*, or *CDR1C*, respectively (Fig. 4B). Disruption of each of these ABC transporter genes alone, or the combination of *CDR1C* with either *CDR1A* or *CDR1B* had no effect on manogepix MIC. Further, the disruption of all three transporter genes decreased manogepix MIC by only one dilution, suggesting there are additional *TAC1* target genes contributing to *TAC1*-mediated reduced manogepix susceptibility.

### A mutation in *PDR1* that confers resistance to fluconazole in *C. glabrata* confers reduced susceptibility to manogepix

**Fig 5:**
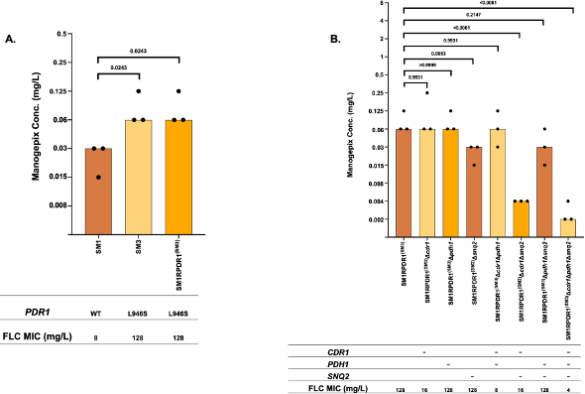
Manogepix MIC for (**A**) *C. glabrata* clinical isolate matched set SM1 and SM3 and SM1 derivative strain harboring *PDR1* allele from SM3, and (**B**) SM1 derivative strain harboring *PDR1* allele from SM3 and its derivative strains in which *CDR1*, *PDH1*, and *SNQ2* have been knocked out individually or in combination. Each bar in the graph represents the modal MIC value, and each dot represents the measurements from three independent assays.

To determine if *PDR1* activating mutations that confer fluconazole resistance in *C. glabrata* influence susceptibility to manogepix, we first measured manogepix susceptibilities in susceptible isolate SM1, its matched fluconazole-resistant isolate SM3, which carries a *PDR1*^L946S^ mutation, and a derivative of SM1 engineered to carry the *PDR1* allele from isolate SM3 (Fig. 5A). Both SM3 and the SM1 derivative carrying the *PDR1*^SM3^ allele exhibited a one-dilution increase in manogepix MIC to 0.06 µg/mL as compared with SM1 with an MIC of 0.03 µg/mL. These findings indicate that activating mutations in *PDR1* that confer fluconazole resistance in *C. glabrata* likewise result in reduced susceptibility to manogepix.

### *SNQ2* and to a lesser extent *CDR1* and *PDH1* are drivers of reduced susceptibility to manogepix in *C. glabrata*

The *PDR1*^L946S^ mutation in isolate SM3 has been shown to drive fluconazole resistance through the upregulation of the genes encoding the Cdr1, Pdh1, and Snq2 ABC transporters (23). We therefore measured manogepix susceptibilities in strains derived from the *PDR1*^SM3^ derivative of isolate SM1 that lack *CDR1*, *PDH1*, and *SNQ2* (Fig. 5B). Deletion of either *CDR1* or *PDH1* alone in this strain had no effect on manogepix MIC, whereas deletion of *SNQ2* increased susceptibility by one dilution from 0.06 µg/mL to 0.03 µg/mL. Deletion of both *CDR1* and *PDH1* had no effect (MIC 0.06 µg/mL), deletion of both *CDR1* and *SNQ2* together by four dilutions to 0.004 µg/mL, and deletion of all three transporters together by five dilutions to 0.002 µg/mL. These data indicate that *SNQ2* is the predominant driver of reduced susceptibility of manogepix due to this *PDR1* mutation.

## DISCUSSION

Fosmanogepix is among several antifungal agents currently in late stages of development and has shown promise in a phase II study of non-neutropenic patients with candidemia (24). Promising results have also been reported in the treatment of a limited number of ICU patients with *C. auris* candidemia. While it has generally shown potent *in vitro* activity against *Candida* species, a correlation to fluconazole susceptibilities has been observed in some studies (7, 8). Our findings suggest that this correlation might be explained by mutations in the transcription factors that drive overexpression of ABC transporters in these species.

A *TAC1B*^N865D^ mutation was recently implicated in decreased manogepix susceptibility in a laboratory-evolved clade I *C. auris* strain (17). This strain exhibited increased *CDR1* expression, and loss of either *CDR1* or *TAC1B* conferred increased susceptibility. In the present study, we observed a similar reduction in manogepix susceptibility in fluconazole-resistant isolates and strains harboring different *TAC1B* mutations. In addition to *CDR1*, these mutations have been shown to drive increased expression of *MDR1,* which encodes a MFS transporter involved in fluconazole resistance. We found that changes in manogepix susceptibility due to these *TAC1B* mutations were due to the overexpression of *CDR1*, not *MDR1*. Unlike mutations identified in fluconazole-resistant isolates, the *TAC1B*^N865D^ mutation generated in manogepix-evolved strains resulted in increased *CDR1* expression but not *MDR1* expression, suggesting that manogepix may preferentially select for *TAC1B* mutations that selectively regulate *CDR1* expression.

We also found that mutations in the zinc cluster transcription factor *TAC1* in *C. albicans* and *C. parapsilosis* that contribute to fluconazole resistance likewise result in decreased susceptibility to manogepix. In *C. albicans*, Tac1 has been shown to upregulate, via activating mutations or in response to inducers, such as fluphenazine, the expression of the ABC transporter genes *CDR1* and *CDR2* and contribute to fluconazole resistance (10). It is likely that the reduced susceptibility to manogepix we observed in isolates and strains carrying *TAC1* mutations is due to the overexpression of one or both of these transporters. However, one limitation of our study is the lack of *CDR1* or *CDR2* deletion mutants within our strain collection, limiting our ability to test this hypothesis. The *TAC1*^G650E^ mutation in *C. parapsilosis* conferred reduced susceptibility, due in part to the Cdr1A and Cdr1C transporters, as disruption of these Cdr genes in the presence of this *TAC1* mutation conferred increased susceptibility. However, as disruption of *CDR1A*, *CDR1B*, and *CDR1C* together did not restore susceptibility to the level observed when the *TAC1*^G650E^ mutation was corrected to the wild-type sequence, it is likely that other Tac1-regulated genes, such as those encoding other transporters, contribute to this phenotype.

In *C. glabrata*, *PDR1* regulates the multidrug-resistant ABC transporter genes *CDR1*, *PDH1*, and *SNQ2*, all of which have been shown to contribute to fluconazole resistance. Importantly, fluconazole resistance in *C. glabrata* is almost exclusively due to such mutations. We found that the *PDR1*^L946S^ mutation, which confers fluconazole resistance in a clinical *C. glabrata* isolate, also confers reduced susceptibility to manogepix. This was dependent in large part on *SNQ2*, but loss of *SNQ2*, *CDR1*, and *PDH1* in combination conferred hypersusceptibility to manogepix, revealing a possible strategy for sensitizing *C. glabrata* to this antifungal.

Our findings demonstrate that, like the *TAC1B*^N865D^ mutation leading to the substitution conferring reduced manogepix susceptibility in the *C. auris* manogepix-evolved laboratory strain, mutations in this and similar transcription factor genes that confer fluconazole resistance in clinical isolates of multiple *Candida* species likewise confer reduced manogepix susceptibility. This may explain previously observed correlations between fluconazole and manogepix susceptibilities and may have clinical implications for treating fluconazole-resistant isolates that harbor such mutations.

## MATERIALS AND METHODS

### Strain and isolate growth conditions

The strains and isolates used in this study are listed in Table 1 and were routinely propagated in YPD (1% yeast extract, 2% peptone, 2% dextrose) at 30°C for all species except for *C. auris* (35°C) and stored in 40% glycerol at −80°C.

**TABLE 1:** Strains and clinical isolates used in this study^*a*^

Strain/Isolate Name	Relevant genotype	Reference
*Candida auris* strains and isolates		
Kw2999	*TAC1B* ^A640V^	25
1c	*TAC1B* ^WT^	13
1cA640V-1	*TAC1B* ^A640V^	13
1cA657V-10A1	*TAC1B* ^A657V^	13
1cADdel-10A	*TAC1B* ^F862_N866del^	13
2999cdr1-16A	*TAC1B*^A640V^/*CDR1*^S31*^	13
1c-cdr1-6A	*TAC1B*^WT^/*CDR1*^S31*^	13
1cA640Vcdr1-4B	*TAC1B*^A640V^/*CDR1*^S31*^	13
1cA657Vcdr1-22A	*TAC1B*^A657V^/*CDR1*^S31*^	13
1cADdelcdr1-8A	*TAC1B*^F862_N866del^/*CDR1*^S31*^	13
2999mdr1-12A	*TAC1B*^A640V^/*MDR1*^S5*^	13
1c-mdr1-8A	*TAC1B*^WT^ /*MDR1*^S5*^	13
1cA640Vmdr1-8A	*TAC1B*^A640V^ /*MDR1*^S5*^	13
1cA657Vmdr1-7A	*TAC1B*^A657V^ /*MDR1*^S5*^	13
1cADdel-mdr1-9A	*TAC1B*^F862_N866del^ /*MDR1*^S5*^	13
2999cdr1-mdr1-7A	*TAC1B*^A640V^/*CDR1*^S31*^/*MDR1*^S5*^	13
1c-cdr1-mdr1-12A	*TAC1B*^WT^/*CDR1*^S31*^/*MDR1*^S5*^	13
1cA640Vcdr1-mdr1-7A	*TAC1B*^A640V^/*CDR1*^S31*^/*MDR1*^S5*^	13
1cA657Vcdr1-mdr1-1A	*TAC1B*^A657V^/*CDR1*^S31*^/*MDR1*^S5*^	13
1cADdel-cdr1-mdr1-3A	*TAC1B*^F862_N866del^/*CDR1*^S31*^/*MDR1*^S5*^	13
AR0387	*TAC1B* ^WT^	CDC clinical isolate
AR0387_TAC1B^A640V^	*TAC1B* ^A640V^	12
AR0390	*TAC1B* ^A640V^	CDC clinical isolate
AR0390_TAC1B^WT^	*TAC1B* ^WT^	12
*Candida albicans* strains and isolates		
Gu4	*TAC1*^WT^/*TAC1*^WT^	18
Gu5	*TAC1*^G980E^/*TAC1*^G980E^	18,19
C43	*TAC1*^WT^/*TAC1*^WT^	20
C56	*TAC1*^N977D^/*TAC1*^N977D^	20
TW3	*TAC1*^WT^/*TAC1*^WT^	21
TW17	*TAC1*^A736V^/*TAC1*^Δ962-969^	21
5457	*TAC1-1*^WT^/*TAC1-2*^WT^	22
5674	*TAC1-1*^N972D^/*TAC1-2*^N972D^	22
SC5314	*TAC1*^WT^/*TAC1*^WT^	26
SZY84	Δ*tac1*/*TAC1-2*^WT^ from 5457	22
SZY86	Δ*tac1*/*TAC1-2*^N972D^ from 5674	22
Candida parapsilosis strains and isolates		
Cp35	*TAC1*^G650E^/*TAC1*^G650E^	27
Cp35-CpTAC1WT	*TAC1*^WT^/*TAC1*^WT^	11
Cp35-1A	*TAC1*^G650E^/*TAC1*^G650E^, Δ*cdr1*/ Δ*cdr1*	11
Cp35-1B	*TAC1*^G650E^/*TAC1*^G650E^, Δ*cdr1b*/ Δ*cdr1b*	11
Cp35-1C	*TAC1*^G650E^/*TAC1*^G650E^, Δ*cdr1c*/ Δ*cdr1c*	11
Cp35-1A1C	*TAC1*^G650E^/*TAC1*^G650E^, Δ*cdr1*/ Δ*cdr1*, Δ*cdr1c*/ Δ*cdr1c*	11
Cp35-1B1C	*TAC1*^G650E^/*TAC1*^G650E^, Δ*cdr1b*/ Δ*cdr1b*, Δ*cdr1c*/ Δ*cdr1c*	11
Cp35-1A1B1C	*TAC1*^G650E^/*TAC1*^G650E^, Δ*cdr1*/ Δ*cdr1*, Δ*cdr1b*/ Δ*cdr1b*, Δ*cdr1c*/ Δ*cdr1c*	11
*Candida glabrata* strains and isolates		
SM1	*PDR1* ^WT^	28
SM3	*PDR1* ^L946S^	28
SM1RPDR1(SM3)	Δ*pdr1*::FRT- *PDR1*^L946S^	16
SM1RPDR1(SM3)Δ*cdr1*	Δ*pdr1*::FRT- *PDR1*^L946S^ Δ*cdr1*	16
SM1RPDR1(SM3) Δ*pdh1*	Δ*pdr1*::FRT- *PDR1*^L946S^ Δ*pdh1*	16
SM1RPDR1(SM3) Δ*snq2*	Δ*pdr1*::FRT- *PDR1*^L946S^ Δ*snq2*	16
SM1RPDR1(SM3) Δ*cdr1*Δ*pdh1*	Δ*pdr1*::FRT- *PDR1*^L946S^ Δ*cdr1*Δ*pdh1*	16
SM1RPDR1(SM3) Δ*cdr1*Δ*snq2*	Δ*pdr1*::FRT- *PDR1*^L946S^ Δ*cdr1*Δ*snq2*	16
SM1RPDR1(SM3) Δ*pdh1*Δ*snq2*	Δ*pdr1*::FRT- *PDR1*^L946S^ Δ*pdh1*Δ*snq2*	16
SM1RPDR1(SM3) Δ*cdr1*Δ*pdh1*Δ*snq2*	Δ*pdr1*::FRT- *PDR1*^L946S^ Δ*cdr1*Δ*pdh1*Δ*snq2*	16

^*a*^ An asterisk (*) indicates introduction of a premature stop codon at the designated residue.

### Broth microdilution assays

Antifungal susceptibility testing was performed by broth microdilution according to the methods published in the CLSI M27 standard (29, 30). Manogepix stocks were prepared in DMSO with further dilutions in RPMI-1640 buffered with 0.165M MOPS (pH 7.0). The concentration range of manogepix that was tested was 0.002 to 1 µg/mL, and the final DMSO concentration per well was 1% v/v. Trays were incubated at 35°C for 24 h, and the manogepix MIC was read as the lowest concentration that resulted in at least 50% inhibition of growth compared with the drug-free growth control well. Each isolate was tested on three separate days. *Candida parapsilosis* ATCC 22019 and *Candida albicans* ATCC 90028 served as the quality control isolates, as recommended by CLSI, and were included on each day of testing.

## AFTER EPUB

*[This article was published on 23 July 2025 with an error in the Discussion section. The Discussion section was corrected in the current version, posted on 4 August 2025.]*

## References

[1] Denning DW. 2024. Global incidence and mortality of severe fungal disease. Lancet Infect Dis 24:e428–e438. doi:10.1016/S1473-3099(23)00692-8 38224705

[2] Pappas PG, Kauffman CA, Andes DR, Clancy CJ, Marr KA, Ostrosky-Zeichner L, Reboli AC, Schuster MG, Vazquez JA, Walsh TJ, Zaoutis TE, Sobel JD. 2016. Clinical practice guideline for the management of candidiasis: 2016 update by the infectious diseases society of America. Clin Infect Dis 62:e1–50. doi:10.1093/cid/civ933 26679628 PMC4725385

[3] Lyman M, Forsberg K, Sexton DJ. 2023. Worsening spread of Candida auris in the United States, 2019 to 2021. Ann Intern Med 176:489–495. doi:10.7326/M22-3469 36940442 PMC11307313

[4] Lockhart SR, Chowdhary A, Gold JAW. 2023. The rapid emergence of antifungal-resistant human-pathogenic fungi. Nat Rev Microbiol 21:818–832. doi:10.1038/s41579-023-00960-9 37648790 PMC10859884

[5] Benedict K, Tsay SV, Bartoces MG, Vallabhaneni S, Jackson BR, Hicks LA. 2021. Outpatient antifungal prescribing patterns in the United States, 2018. ASHE 1:e68. doi:10.1017/ash.2021.201 PMC9336187 35910521

[6] Lamoth F, Lewis RE, Kontoyiannis DP. 2022. Investigational antifungal agents for invasive mycoses: a clinical perspective. Clin Infect Dis 75:534–544. doi:10.1093/cid/ciab1070 34986246

[7] Arendrup MC, Jørgensen KM. 2020. Manogepix (APX001A) displays potent in vitro activity against human pathogenic yeast, but with an unexpected correlation to fluconazole MICs . Antimicrob Agents Chemother 64:e00429–20. doi:10.1128/AAC.00429-20 32366708 PMC7318001

[8] Arendrup MC, Chowdhary A, Astvad KMT, Jørgensen KM. 2018. APX001A in vitro activity against contemporary blood isolates and candida auris determined by the EUCAST reference method . Antimicrob Agents Chemother 62:e01225–18. doi:10.1128/AAC.01225-18 30104264 PMC6153824

[9] Liston SD, Whitesell L, Kapoor M, Shaw KJ, Cowen LE. 2020. Enhanced efflux pump expression in Candida mutants results in decreased manogepix susceptibility. Antimicrob Agents Chemother 64:e00261-20. doi:10.1128/AAC.00261-20 32179530 PMC7179633

[10] Coste AT, Karababa M, Ischer F, Bille J, Sanglard D. 2004. TAC1, transcriptional activator of CDR genes, is a new transcription factor involved in the regulation of Candida albicans ABC transporters CDR1 and CDR2. Eukaryot Cell 3:1639–1652. doi:10.1128/EC.3.6.1639-1652.2004 15590837 PMC539021

[11] Doorley LA, Barker KS, Zhang Q, Rybak JM, Rogers PD. 2023. Mutations in TAC1 and ERG11 are major drivers of triazole antifungal resistance in clinical isolates of Candida parapsilosis. Clin Microbiol Infect 29:1602. doi:10.1016/j.cmi.2023.08.030 37666448

[12] Rybak JM, Muñoz JF, Barker KS, Parker JE, Esquivel BD, Berkow EL, Lockhart SR, Gade L, Palmer GE, White TC, Kelly SL, Cuomo CA, Rogers PD. 2020. Mutations in TAC1B: a novel genetic determinant of clinical fluconazole resistance in Candida auris. MBio 11:e00365-20. doi:10.1128/mBio.00365-20 32398311 PMC7218281

[13] Barker KS, Santana DJ, Zhang Q, Peters TL, Rybak J, Morschhauser J, Cuomo CA, Rogers PD. 2025. Mutations in TAC1B drive CDR1 and MDR1 expression and azole resistance in C. auris. bioRxiv. doi:10.1101/2025.02.11.637698 PMC12486835 40824653

[14] Torelli R, Posteraro B, Ferrari S, La Sorda M, Fadda G, Sanglard D, Sanguinetti M. 2008. The ATP-binding cassette transporter-encoding gene CgSNQ2 is contributing to the CgPDR1-dependent azole resistance of Candida glabrata. Mol Microbiol 68:186–201. doi:10.1111/j.1365-2958.2008.06143.x 18312269

[15] Vermitsky JP, Earhart KD, Smith WL, Homayouni R, Edlind TD, Rogers PD. 2006. Pdr1 regulates multidrug resistance in Candida glabrata: gene disruption and genome-wide expression studies. Mol Microbiol 61:704–722. doi:10.1111/j.1365-2958.2006.05235.x 16803598

[16] Whaley SG, Zhang Q, Caudle KE, Rogers PD. 2018. Relative contribution of the ABC transporters Cdr1, Pdh1, and Snq2 to azole resistance in Candida glabrata. Antimicrob Agents Chemother 62:e01070-18. doi:10.1128/AAC.01070-18 30038038 PMC6153852

[17] Hirayama T, Miyazaki T, Tanaka R, Kitahori N, Yoshida M, Takeda K, Ide S, Iwanaga N, Tashiro M, Takazono T, Izumikawa K, Yanagihara K, Makimura K, Tsukamoto K, Mukae H. 2025. TAC1b mutation in Candida auris decreases manogepix susceptibility owing to increased CDR1 expression. Antimicrob Agents Chemother:e0150824. doi:10.1128/AAC.01508-24 39692503 PMC11823642

[18] Franz R, Ruhnke M, Morschhäuser J. 1999. Molecular aspects of fluconazole resistance development in Candida albicans. Mycoses 42:453–458. doi:10.1046/j.1439-0507.1999.00498.x 10546486

[19] Popp C, Hampe IAI, Hertlein T, Ohlsen K, Rogers PD, Morschhäuser J. 2017. Competitive fitness of fluconazole-resistant clinical Candida albicans strains. Antimicrob Agents Chemother 61:e00584-17. doi:10.1128/AAC.00584-17 28461316 PMC5487674

[20] Sanglard D, Kuchler K, Ischer F, Pagani JL, Monod M, Bille J. 1995. Mechanisms of resistance to azole antifungal agents in Candida albicans isolates from AIDS patients involve specific multidrug transporters. Antimicrob Agents Chemother 39:2378–2386. doi:10.1128/AAC.39.11.2378 8585712 PMC162951

[21] White TC. 1997. Increased mRNA levels of ERG16, CDR, and MDR1 correlate with increases in azole resistance in Candida albicans isolates from a patient infected with human immunodeficiency virus. Antimicrob Agents Chemother 41:1482–1487. doi:10.1128/AAC.41.7.1482 9210670 PMC163944

[22] Znaidi S, De Deken X, Weber S, Rigby T, Nantel A, Raymond M. 2007. The zinc cluster transcription factor Tac1p regulates PDR16 expression in Candida albicans. Mol Microbiol 66:440–452. doi:10.1111/j.1365-2958.2007.05931.x 17897373

[23] Caudle KE, Barker KS, Wiederhold NP, Xu L, Homayouni R, Rogers PD. 2011. Genomewide expression profile analysis of the Candida glabrata Pdr1 regulon. Eukaryot Cell 10:373–383. doi:10.1128/EC.00073-10 21193550 PMC3067479

[24] Pappas PG, Vazquez JA, Oren I, Rahav G, Aoun M, Bulpa P, Ben-Ami R, Ferrer R, Mccarty T, Thompson GR, Schlamm H, Bien PA, Barbat SH, Wedel P, Oborska I, Tawadrous M, Hodges MR. 2023. Clinical safety and efficacy of novel antifungal, fosmanogepix, for the treatment of candidaemia: results from a Phase 2 trial. J Antimicrob Chemother 78:2471–2480. doi:10.1093/jac/dkad256 37596890 PMC10545531

[25] Ahmad S, Khan Z, Al-Sweih N, Alfouzan W, Joseph L. 2020. Candida auris in various hospitals across Kuwait and their susceptibility and molecular basis of resistance to antifungal drugs. Mycoses 63:104–112. doi:10.1111/myc.13022 31618799

[26] Gillum AM, Tsay EY, Kirsch DR. 1984. Isolation of the Candida albicans gene for orotidine-5’-phosphate decarboxylase by complementation of S. cerevisiae ura3 and E. coli pyrF mutations. Mol Gen Genet 198:179–182. doi:10.1007/BF00328721 6394964

[27] Berkow EL, Manigaba K, Parker JE, Barker KS, Kelly SL, Rogers PD. 2015. Multidrug transporters and alterations in sterol biosynthesis contribute to azole antifungal resistance in Candida parapsilosis. Antimicrob Agents Chemother 59:5942–5950. doi:10.1128/AAC.01358-15 26169412 PMC4576097

[28] Magill SS, Shields C, Sears CL, Choti M, Merz WG. 2006. Triazole cross-resistance among Candida spp.: case report, occurrence among bloodstream isolates, and implications for antifungal therapy. J Clin Microbiol 44:529–535. doi:10.1128/JCM.44.2.529-535.2006 16455909 PMC1392670

[29] CLSI. 2017. Reference method for broth dilution antifungal susceptibility testing of yeasts. In CLSI Standard M27, 4th ed. Clinical and Laboratory Standards Institute, Wayne, Pennsylvania.

[30] CLSI. 2022. Performance standards for antifungal susceptibility testing of yeasts. In CLSI supplement M27M44S, 3rd ed. Clinical and Laboratory Standards Institute.

